# Health‐related quality‐of‐life analyses from a multicenter, randomized, double‐blind phase 2 study of patients with differentiated thyroid cancer treated with lenvatinib 18 or 24 mg/day

**DOI:** 10.1002/cam4.5308

**Published:** 2022-12-04

**Authors:** Matthew H. Taylor, Sophie Leboulleux, Yury Panaseykin, Bhavana Konda, Christelle de La Fouchardiere, Brett G. M. Hughes, Andrew G. Gianoukakis, Young Joo Park, Ilia Romanov, Monika K. Krzyzanowska, Diana Garbinsky, Bintu Sherif, Jie Janice Pan, Terri A. Binder, Nicholas Sauter, Ran Xie, Marcia S. Brose

**Affiliations:** ^1^ Earle A. Chiles Research Institute, Providence Portland Medical Center Portland Oregon USA; ^2^ Department of Nuclear Medicine and Endocrine Oncology Gustave Roussy and University Paris Saclay Villejuif France; ^3^ A. Tsyb Medical Radiological Research Center, branch of the NMRС of Radiology Obninsk Russian Federation; ^4^ Division of Medical Oncology The Ohio State University Comprehensive Cancer Center Ohio Columbus USA; ^5^ Medical Oncology Department Centre Léon Bérard Lyon France; ^6^ Department of Cancer Care Services Royal Brisbane and Women's Hospital, University of Queensland Queensland Australia; ^7^ The Lundquist Institute at Harbor‐UCLA Medical Center, David Geffen School of Medicine at UCLA California Los Angeles/Torrance USA; ^8^ Department of Internal Medicine Seoul National University College of Medicine Seoul Republic of Korea; ^9^ Department of Head & Neck Tumors N.N. Blokhin Russian Cancer Research Center Moscow Russian Federation; ^10^ Department of Medical Oncology & Hematology Princess Margaret Cancer Centre Ontario Toronto Canada; ^11^ RTI Health Solutions Research Triangle Park North Carolina USA; ^12^ Global Value and Access (GV&A) Oncology, Eisai Inc. New Jersey Nutley USA; ^13^ Oncology Clinical Research Eisai Inc. New Jersey Nutley USA; ^14^ Biostatistics, Eisai Inc. New Jersey Nutley USA; ^15^ Department of Medical Oncology Sidney Kimmel Cancer Center, Jefferson University (previous affiliation: Department of Otorhinolaryngology: Head and Neck Surgery, Abramson Cancer Center, University of Pennsylvania) Pennsylvania Philadelphia USA

**Keywords:** dose intensity, HRQoL, kinase inhibitor, lenvatinib, quality of life, radioiodine‐refractory differentiated thyroid cancer

## Abstract

**Background:**

In the phase 2 double‐blind Study 211, a starting dose of lenvatinib 18 mg/day was compared with the approved starting dose of 24 mg/day in patients with radioiodine‐refractory differentiated thyroid cancer (RR‐DTC). Predefined criteria for noninferiority for efficacy in the 18 mg arm were not met; safety was similar in both arms. Impact of lenvatinib treatment on health‐related quality‐of‐life (HRQoL) was a secondary endpoint of Study 211.

**Methods:**

Patients with RR‐DTC were randomly assigned to a blinded starting dose of lenvatinib 18 mg/day or 24 mg/day. HRQoL was assessed at baseline, every 8 weeks until Week 24, then every 16 weeks, and at the off‐treatment visit, using the EQ‐5D‐3L and FACT‐G instruments. Completion and compliance rates, mean change from baseline, and times to first and definitive deterioration were evaluated.

**Results:**

Baseline EQ‐5D and FACT‐G scores, and overall changes from baseline, were comparable between patients in the lenvatinib 18 mg/day (*n* = 77) and 24 mg/day arms (*n* = 75). For the 18 mg versus 24 mg arms, least squares mean differences were −0.42 (95% CI −4.88, 4.03) for EQ‐5D‐VAS and 0.47 (95% CI −3.45, 4.39) for FACT‐G total. Time to first deterioration did not significantly favor either arm; EQ‐5D‐VAS HR [18 mg/24 mg] 0.93 (95% CI 0.61–1.40), EQ‐5D‐HUI HR [18 mg/24 mg] 0.68 (95% CI 0.44–1.05), FACT‐G total HR [18 mg/24 mg] 0.73 (95% CI 0.48–1.12). Time to definitive deterioration did not significantly favor either arm, though EQ‐5D‐VAS showed a trend in favor of the 24 mg arm (HR [18 mg/24 mg] 1.72; 95% CI 0.99–3.01); EQ‐5D‐HUI HR [18 mg/24 mg] was 0.96 (95% CI 0.57–1.63), FACT‐G total HR [18 mg/24 mg] was 0.72 (95% CI 0.43–1.21).

**Conclusions:**

In Study 211, HRQoL for patients in the lenvatinib 18 mg/day arm was not statistically different from that of patients in the 24 mg/day arm. These data further support the use of the approved lenvatinib starting dose of 24 mg/day in patients with RR‐DTC.

**ClinicalTrials.gov Number:**

NCT02702388.

## Graphical abstract

Evaluation of the impact of lenvatinib treatment on health‐related quality‐of‐life was a secondary objective of the phase 2 double‐blinded Study 211. This study compared a starting dose of lenvatinib 18 mg/day with the approved starting dose of 24 mg/day in patients with radioiodine‐refractory differentiated thyroid cancer. Health‐related quality‐of‐life of patients in the 18 mg/day arm was not statistically different from that of patients in the 24 mg/day arm. These data, combined with results from the primary analysis (where the lenvatinib starting dose of 18 mg/day did not demonstrate noninferiority to the approved starting dose of lenvatinib 24 mg/day), support the use of the approved lenvatinib starting dose of 24 mg/day in patients with radioiodine‐refractory differentiated thyroid cancer.

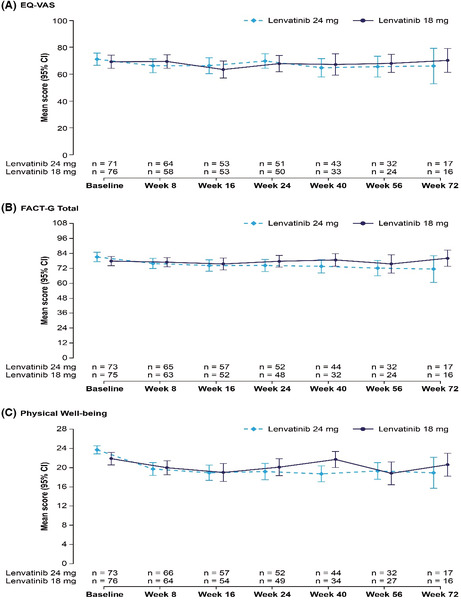

## 1 INTRODUCTION

The ability to effectively treat cancer while maintaining patients' health‐related quality of life (HRQoL) is critically important to maximize therapeutic benefits. Cancer diagnoses impair patients' HRQoL both during and after treatment^1^, ^2^, ^3^ as HRQoL is impacted by disease progression^4^, ^5^ and by anticancer therapies. Among patients with radioiodine‐refractory differentiated thyroid cancer (RR‐DTC), first‐line systemic treatment recommendations include lenvatinib, which is considered a preferred treatment by the National Comprehensive Cancer Network and the European Society for Medical Oncology.^6^, ^7^ Adverse events (AEs) that can negatively affect quality of life (QoL) and are commonly associated with kinase inhibitors such as lenvatinib include fatigue, diarrhea, anorexia, weight loss, rash, nausea, and musculoskeletal pain.^8^, ^9^, ^10^

In the SELECT study, lenvatinib 24 mg/day demonstrated superior efficacy compared with placebo in patients with RR‐DTC.^11^ However, AEs led to dose interruption and reduction in 82.4% and 67.8% of patients treated with lenvatinib, respectively,^11^ and raised the question of whether a lower starting dose of lenvatinib would benefit patients' QoL while providing equivalent efficacy.

Study 211 was designed to determine if a lower starting dose of lenvatinib (18 mg/day) would provide comparable efficacy and an improved safety profile versus the approved starting dose of 24 mg/day in patients with RR‐DTC.^12^ In Study 211, the objective response rate (ORR) as of 24 weeks, as assessed by investigators using Response Evaluation Criteria in Solid Tumors version 1.1 (RECIST v1.1), was 40.3% in the lenvatinib 18 mg/day starting dose arm and 57.3% in the 24 mg/day starting dose arm (odds ratio 0.50, 95% confidence interval [CI] 0.26–0.96). This difference in ORR as of Week 24 was clinically relevant and was maintained for the entire study duration. Thus, the 18 mg/day starting dose failed to demonstrate non‐inferiority to the approved starting dose of 24 mg/day. Although the study was not powered to show a difference between the treatment arms for progression‐free survival (PFS), results appeared to favor the lenvatinib 24 mg/day starting dose (median not reached [95% CI 22.1–not estimable]) versus the 18 mg/day starting dose (median 24.4 months [95% CI 14.7–not estimable]; hazard ratio (HR) 18 mg/24 mg 1.44, 95% CI 0.76–2.74). Moreover, the primary safety endpoint, incidence of treatment‐emergent AEs (TEAEs) of grade ≥3 as of Week 24, was similar between the two arms; 57.1% in the lenvatinib 18 mg/day starting dose arm and 61.3% in the 24 mg/day starting dose arm, with a calculated difference of −4.2% (95% CI: −19.8, 11.4) using asymptotic normal approximation. In addition, the safety profile of the two arms was consistent with the known safety profile for lenvatinib in patients with RR‐DTC.^12^ These efficacy and safety data support the use of the approved lenvatinib 24 mg/day starting dose. The HRQoL data described herein will provide further evidence to support the approved starting dose.

Few data exist on HRQoL in patients with RR‐DTC receiving kinase inhibitor therapies. Because real‐world analyses typically include few patients and prospective controlled clinical trial data are lacking, the effect of kinase inhibitor treatment, and lenvatinib specifically, on HRQoL in patients with RR‐DTC is an area that requires additional research.^13^ Assessment of HRQoL was a secondary objective of Study 211, and data were prospectively collected in this trial. Herein, we report comparisons of HRQoL patient‐reported outcomes between the lenvatinib 18 mg/day and 24 mg/day starting doses.

## 2 METHODS

### 2.1 Study design and patients

Eligibility details of this phase 2 multicenter, randomized, double‐blind trial have been previously published.^12^ Briefly, patients were aged ≥18 years at the time of informed consent and had confirmed RR‐DTC with measurable disease per RECIST v1.1. Patients also had an Eastern Cooperative Oncology Group performance status (ECOG PS) of ≤2 and adequate organ function; patients who had received ≥2 previous vascular endothelial growth factor/vascular endothelial growth factor receptor‐targeted therapies were excluded.

Written informed consent was provided by all patients before undergoing any study‐specific procedures. The study protocol was approved by relevant institutional review boards and was conducted in accordance with the principles of the World Medical Association Declaration of Helsinki.

Patients were randomly assigned (1:1) to receive a starting dose of lenvatinib 18 mg/day or 24 mg/day. Randomization of masked lenvatinib dose packs was performed centrally by an interactive voice‐ and webresponse system. Double‐blind lenvatinib dosing was used to minimize patient and investigator bias. Patients were stratified by age (≤ 65 years vs. > 65 years) and ECOG PS (0 vs. 1 or 2).

A secondary objective of Study 211 was to evaluate the effect of lenvatinib treatment on HRQoL as measured by the EuroQol 5 Dimensions 3 Levels (EQ‐5D‐3L) and Functional Assessment of Cancer Therapy‐General (FACT‐G). HRQoL was assessed at baseline, every 8 weeks until Week 24, then every 16 weeks, and at the off‐treatment visit.

### 2.2 Patient‐reported outcome instruments

The EQ‐5D‐3L is divided into the EQ‐5D descriptive system, consisting of 5 dimensions (mobility, self‐care, usual activities, pain/discomfort, anxiety/depression), and the EQ‐5D visual analog scale (VAS).^14^ The EQ‐5D Health Utilities Index (HUI) is derived from the dimensions of the EQ‐5D using country‐specific preference weights. The FACT‐G measures the effect of cancer treatment on QoL in four subscales (physical, social/family, emotional, and functional well‐being), which are summed to get a total score.^15^ See the Data S1 for additional details.

### 2.3 Statistical analyses

Statistical tests and CIs were 2‐sided and had an associated alpha level of 0.05. No statistical hypotheses were prespecified for HRQoL outcomes. No adjustments for multiple testing or estimation were used for HRQoL analyses, so all *p* values and CIs are nominal and should be considered descriptive in nature. All analyses were performed using SAS, version 9.4 (SAS Institute, Cary, NC, USA). See the Data S1 for additional details.

#### 2.3.1 Completion and compliance

Completion and compliance rates were summarized for each HRQoL instrument and outcome. An instrument was considered complete if at least one valid scale score could be computed from the available response data. The rate was computed as a percentage of patients in the full analysis set. The compliance rate was the percentage of patients who had completed the instrument among all patients who were expected to complete the instrument at a certain time point (i.e., the number of patients with a valid baseline score who were enrolled in the study and taking the study treatment at that time point).

#### 2.3.2 Longitudinal analysis

To assess the effect of lenvatinib starting dose on EQ‐5D and FACT‐G outcomes, mixed models with random coefficients were fitted using the change from baseline for each HRQoL score as the response variable. The least squares (LS) mean change from baseline for each treatment arm was estimated at each timepoint (not including the off‐treatment assessment), along with an overall LS mean. The difference in LS mean for the lenvatinib 18 mg arm versus the 24 mg arm, along with associated 95% CI and *p* value, was also estimated.

#### 2.3.3 Time to deterioration

Time to deterioration (TTD) was evaluated using the Kaplan–Meier method to estimate the distribution of TTD and median TTD for each treatment arm. Time to first deterioration and time to definitive deterioration (defined in the Data S1) were assessed. The preplanned TTD analyses included those for the EQ‐5D VAS, EQ‐5D HUI, and FACT‐G total score. Comparisons were made between the TTD distributions of each lenvatinib treatment arm using unstratified log‐rank tests. Cox models stratified by the randomization stratification variables were fit for each HRQoL score; HRs and associated 95% CIs were estimated to compare the two treatment arms.

#### 2.3.4 Tumor responder analyses

An exploratory analysis using pooled data from both study arms was conducted to examine the relationship between radiologic tumor responses and HRQoL. Patients were characterized by their best overall radiologic response as responders (complete or partial response) or nonresponders (stable disease, progressive disease, or unknown/not evaluated). Time to first deterioration and time to definitive deterioration were assessed for responders versus nonresponders using similar methods as those described in the TTD section.

## 3 RESULTS

### 3.1 Patient disposition and baseline characteristics

Patients were randomly assigned to the lenvatinib 18 mg starting dose arm (*n* = 77) and 24 mg starting dose arm (*n* = 75). Patient demographics and baseline characteristics have been previously reported and were generally well‐balanced between the treatment arms. The median ages were 66.0 years (range 21–89) and 65.0 years (range 36–92) in the lenvatinib 18 mg and 24 mg arms, respectively. In the lenvatinib 18 mg arm, 48.1% of patients were male and 54.7% of patients were male in the lenvatinib 24 mg arm. Baseline thyroid‐stimulating hormone use and ECOG PS scores were similar between the two treatment arms. Most patients had papillary RR‐DTC (84.0% and 75.3% in the lenvatinib 18 and 24 mg starting dose arms, respectively).^12^

As of the data cutoff date (December 12, 2019), 45.5% and 57.3% of patients in the lenvatinib 18 and 24 mg starting‐dose arms, respectively, were still undergoing treatment. Disease progression was the primary reason for discontinuation in 26.0% and 17.3% of patients in the lenvatinib 18 mg and 24 mg starting‐dose arms, respectively; and AEs were the primary reason for discontinuation in 15.6% of patients in the lenvatinib 18 mg starting‐dose arm, and 13.3% of patients in the 24 mg starting dose arm. All TEAEs that resulted in discontinuation of lenvatinib were each reported by no more than one patient in either treatment arm.

### 3.2 Completion and compliance

At baseline and throughout the assessment period, HRQoL instrument completion and compliance rates were similar for both groups (Table 1). While HRQoL instrument completion rates dropped over time (primarily because of patient discontinuation from treatment due to disease progression, AEs, or other reasons), compliance rates were high at baseline and remained high through Week 56.

**TABLE 1:** HRQoL instrument completion and compliance rates

Parameter	Lenvatinib dose			
	18 mg (*n* = 77)	24 mg (*n* = 75)	18 mg (*n* = 77)	24 mg (*n* = 75)
Completion rates	EQ‐5D‐3L, *n* (%)		FACT‐G, *n* (%)	
Baseline	76 (98.7)	73 (97.3)	76 (98.7)	73 (97.3)
Week 24	52 (67.5)	52 (69.3)	50 (64.9)	53 (70.7)
Week 56	27 (35.1)	32 (42.7)	27 (35.1)	32 (42.7)
Compliance rates	EQ‐5D‐3L, *n* /*m* ^a^ (%)		FACT‐G, *n*/*m* ^a^ (%)	
Baseline	76/77 (98.7)	73/75 (97.3)	76/77 (98.7)	73/75 (97.3)
Week 24	52/56 (92.9)	52/56 (92.9)	50/56 (89.3)	53/56 (94.6)
Week 56	27/28 (96.4)	32/33 (97.0)	27/28 (96.4)	32/33 (97.0)

Abbreviations: EQ‐5D‐3L, EuroQol 5‐dimension 3‐levels scale; FACT‐G, Functional Assessment of Cancer Therapy‐General; HRQoL, health‐related quality of life.

^a^ *n* = the number of patients with a valid score at the specified visit; *m* = number of patients in the full analysis set who were expected to complete an assessment at the specified visit.

### 3.3 Longitudinal change from baseline

**FIGURE 1:**
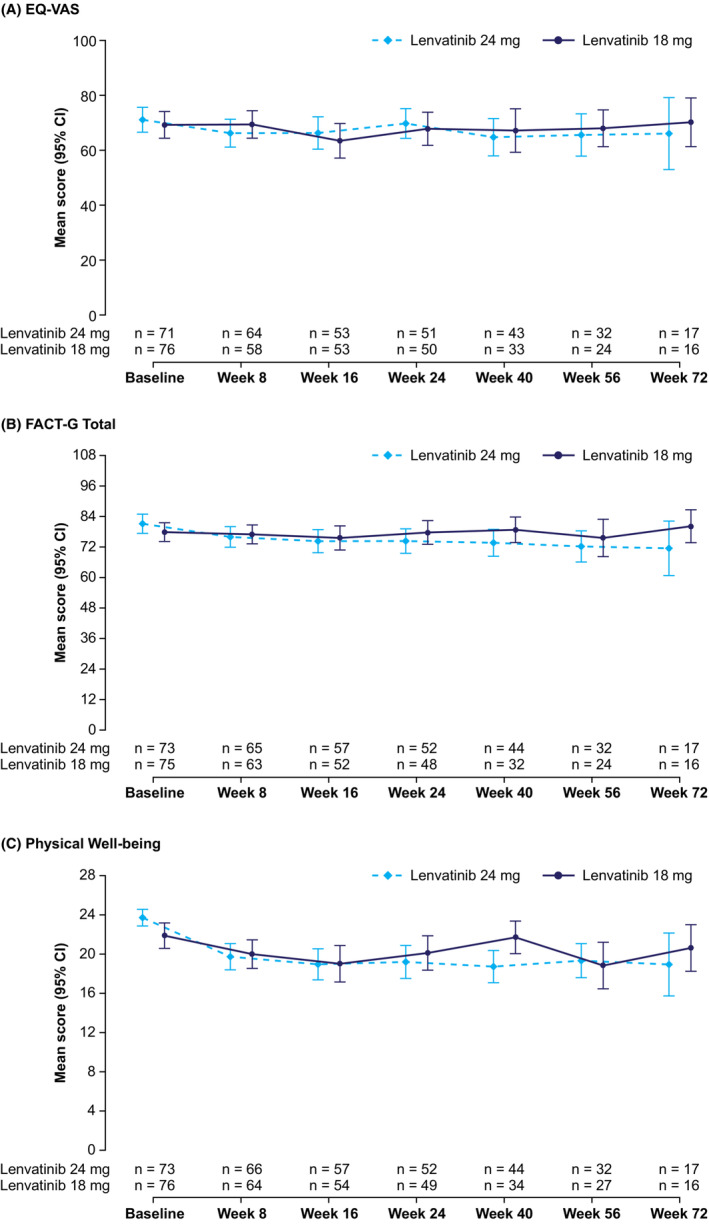
Mean scores over time for (A) EQ‐VAS, (B) FACT‐G total, and (C) FACT‐G physical well‐being functional scale. Scheduled assessments with fewer than 10% of patients still enrolled in each treatment arm are not displayed. CI, confidence interval; EQ‐VAS, EuroQol visual analog scale; FACT‐G, Functional Assessment of Cancer Therapy–General.

The EQ‐5D and FACT‐G scores at baseline were comparable between the treatment arms (Table 2). Baseline EQ‐5D VAS was 69.2 in the lenvatinib 18 mg arm and 71.1 in the lenvatinib 24 mg arm, and the FACT‐G total score was 77.8 in the lenvatinib 18 mg arm and 81.1 in the lenvatinib 24 mg arm. Mean scores remained stable over the assessment period (Figure 1, Figure S1). Overall, mean changes from baseline in HRQoL scores were similar between the lenvatinib 18 mg and 24 mg arms. There were no statistically significant differences in LS mean change between treatment arms for either EQ‐5D or FACT‐G HRQoL scores (Table 3). The LS mean change for the EQ‐5D VAS was −5.68 (standard error [SE] 1.619) in the lenvatinib 18 mg arm and −5.25 (SE 1.601) in the lenvatinib 24 mg arm, a mean difference of −0.42 (95% CI −4.88, 4.03). The LS mean change for the FACT‐G total score was −4.14 (SE 1.438) in the lenvatinib 18 mg arm and −4.61 (SE 1.397) in the lenvatinib 24 mg arm, a mean difference of 0.47 (95% CI −3.45, 4.39).

**TABLE 2:** HRQoL scores at baseline

Scale	HRQoL score, mean (SD)	
	18 mg (*n* = 77)	24 mg (*n* = 75)
EQ‐5D‐3L		
EQ‐VAS	69.2 (21.29)	71.1 (19.12)
HUI	0.8 (0.23)	0.8 (0.17)
FACT‐G		
Total score	77.8 (16.04)	81.1 (16.18)
Physical well‐being	21.9 (5.70)	23.7 (3.64)
Social/family well‐being	21.6 (5.54)	21.6 (6.24)
Emotional well‐being	16.7 (4.15)	17.7 (4.05)
Functional well‐being	17.8 (6.02)	18.1 (6.61)

Abbreviations: EQ‐5D‐3L, EuroQol 5‐dimension 3‐level scale; EQ‐VAS, EuroQol visual analog scale; FACT‐G, Functional Assessment of Cancer Therapy‐General; HRQoL, health‐related quality of life; HUI, health utilities index; SD, standard deviation.

**TABLE 3:** Longitudinal change from baseline in overall least squares mean scores in HRQoL

Scale	LS mean change (SE)		LS mean difference (95% CI)	LS mean difference *p* value
	Lenvatinib 18 mg	Lenvatinib 24 mg	Lenvatinib 18 mg vs. 24 mg	
EQ‐5D‐3L				
VAS	−5.68 (1.619)	−5.25 (1.601)	−0.42 (−4.88, 4.03)	0.8507
HUI	−0.08 (0.018)	−0.06 (0.017)	−0.02 (−0.07, 0.03)	0.4589
FACT‐G				
Total score	−4.14 (1.438)	−4.61 (1.397)	0.47 (−3.45, 4.39)	0.8132
Physical well‐being	−3.13 (0.518)	−3.61 (0.510)	0.48 (−0.95, 1.92)	0.5058
Social/family well‐being	−0.07 (0.525)	0.03 (0.518)	−0.10 (−1.54, 1.34)	0.8886
Emotional well‐being	0.91 (0.323)	0.34 (0.319)	0.57 (−0.32, 1.46)	0.2076
Functional well‐being	−1.56 (0.531)	−1.28 (0.529)	−0.28 (−1.74, 1.19)	0.7076

Abbreviations: CI, confidence interval; EQ‐5D‐3L, EuroQol 5‐dimension 3‐level scale; FACT‐G, Functional Assessment of Cancer Therapy‐General; HRQoL, health‐related quality of life; HUI, health utilities index; LS, least squares; SE, standard error; VAS, visual analog scale.

### 3.4 Time to deterioration

**FIGURE 2:**
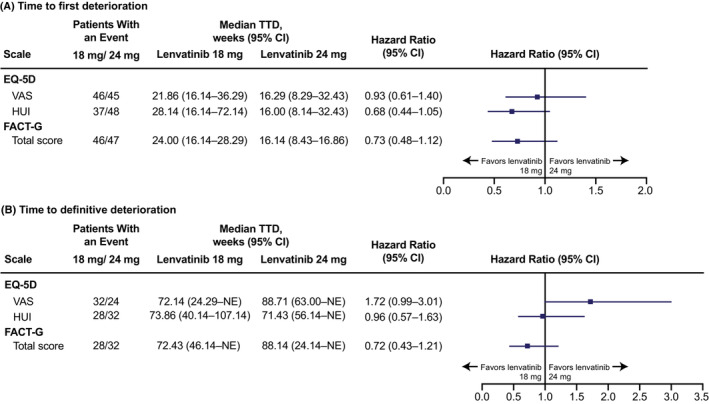
TTD^a^ in patients who received starting doses of lenvatinib 18 mg/day versus 24 mg/day. (A) Time to first deterioration. (B) Time to definitive deterioration. ^a^A deterioration event for a HRQoL outcome was defined as a detrimental change in score relative to baseline that exceeded the MID threshold for decline for that score. MIDs used in this analysis were: decrease of ≥7 points (FACT‐G^19^, ^20^), decrease of ≥0.08 points (HUI^21^), and decrease of ≥7 points (EQ‐VAS^21^). Time to first deterioration was defined as the number of weeks between randomization and the first deterioration event during the treatment period. Time to definitive deterioration was defined as the number of weeks between randomization and the earliest deterioration event during the treatment period with no subsequent recovery above the deterioration threshold. CI, confidence interval; EQ‐5D, EuroQol 5‐dimension scale; FACT‐G, Functional Assessment of Cancer Therapy–General; HUI, health utilities index; MID, minimally important difference; NE, not estimable; TTD, time to deterioration; VAS, visual analog scale.

No significant differences were observed in time to first deterioration between the treatment arms (Figure 2A). Among the 77 and 75 patients in the lenvatinib 18 and 24 mg arms, respectively, first deterioration events were experienced by 46 and 45 patients for the EQ‐5D VAS, 37 and 48 patients for the EQ‐5D HUI, and 46 and 47 patients for the FACT‐G total score. The median time to first deterioration for the EQ‐5D VAS was 21.86 weeks (95% CI 16.14–36.29) for the lenvatinib 18 mg starting dose arm and 16.29 weeks (95% CI 8.29–32.43) for the 24 mg starting dose arm (treatment arm comparison: HR 0.93; 95% CI 0.61–1.40). For the EQ‐5D HUI, the median time to first deterioration was 28.14 weeks (95% CI 16.14–72.14) for the lenvatinib 18 mg starting dose arm and 16.00 weeks (95% CI 8.14–32.43) for the 24 mg starting dose arm (treatment arm comparison: HR 0.68; 95% CI 0.44–1.05). The median time to first deterioration for the FACT‐G total score was 24.00 weeks (95% CI 16.14–28.29) for the lenvatinib 18 mg starting dose arm and 16.14 weeks (95% CI 8.43–16.86) for the 24 mg starting dose arm (treatment arm comparison: HR 0.73; 95% CI 0.48–1.12).

In addition, no significant differences were observed between the treatment arms in time to definitive deterioration (Figure 2B). Definitive deterioration events in the lenvatinib 18 mg and 24 mg arms, respectively, were experienced by 32 and 24 patients for the EQ‐5D VAS, 28 and 32 patients for the EQ‐5D HUI, and 28 and 32 patients for the FACT‐G total score. The treatment arm comparison for time to definitive deterioration for the EQ‐5D VAS showed a trend toward favoring the lenvatinib 24 mg arm (HR 1.72; 95% CI 0.99–3.01). Treatment arm comparisons for time to definitive deterioration for the EQ‐5D HUI and the FACT‐G total score showed no significant differences between arms (EQ‐5D HUI: HR 0.96; 95% CI 0.57–1.63; FACT‐G total score: HR 0.72; 95% CI 0.43–1.21).

### 3.5 Tumor responder analysis by best overall response

**FIGURE 3:**
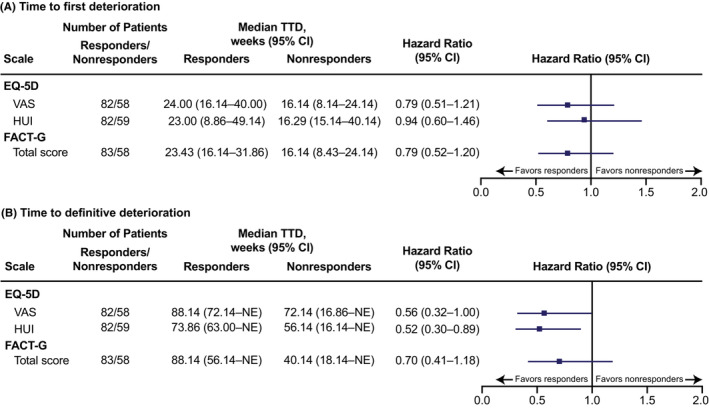
TTD^a^ in radiologic responders versus nonresponders to lenvatinib. (A) Time to first deterioration. (B) Time to definitive deterioration. ^a^A deterioration event for a HRQoL outcome was defined as a detrimental change in score relative to baseline that exceeded the MID threshold for decline for that score. MIDs used in this analysis were: decrease of ≥7 points (FACT‐G^19^, ^20^), decrease of ≥0.08 points (HUI^21^), and decrease of ≥7 points (EQ‐VAS^21^). Time to first deterioration was defined as the number of weeks between randomization and the first deterioration event during the treatment period. Time to definitive deterioration was defined as the number of weeks between randomization and the earliest deterioration event during the treatment period with no subsequent recovery above the deterioration threshold. CI, confidence interval; EQ‐5D, EuroQol 5‐dimension scale; FACT‐G, Functional Assessment of Cancer Therapy–General; HUI, health utilities index; MID, minimally important difference; NE, not estimable; TTD, time to deterioration; VAS, visual analog scale.

There was a numerical, but not statistically significant, trend toward a longer median time to first deterioration among radiologic responders versus nonresponders (as assessed by best overall response) for the EQ‐5D VAS, EQ‐5D HUI, and FACT‐G total score. A similar trend was observed for time to definitive deterioration, though EQ‐5D HUI nominally significantly favored responders (Figure 3).

## 4 DISCUSSION

In this analysis, there were no significant differences in HRQoL between the lenvatinib 18 mg/day and 24 mg/day starting dose arms. Specifically, there were no statistically significant differences in LS mean change between treatment arms for both EQ‐5D and FACT‐G HRQoL scores, time to first deterioration, or time to definitive deterioration.

The blinded nature of this study allowed us to address lenvatinib starting dose selection while minimizing patient and investigator bias. In real‐world practice, some clinicians initiate lenvatinib treatment at doses lower than the approved 24 mg/day starting dose in an attempt to minimize treatment‐emergent AEs (TEAEs) and optimize HRQoL; however, this may prevent patients from receiving the full efficacy benefits of lenvatinib. The Study 211 HRQoL results combined with the primary analysis suggest that use of a lower lenvatinib starting dose may compromise efficacy without improving HRQoL or safety in patients with RR‐DTC.

The HRQoL data from Study 211 are particularly valuable because of the dearth of HRQoL data in patients with thyroid cancer treated with targeted therapies. An analysis that assessed studies that included HRQoL outcomes in patients with thyroid cancer reviewed 94 articles published between 2000 and 2019 and found few studies with data on HRQoL in patients with thyroid cancer treated with kinase inhibitors.^16^ Among patients with RR‐DTC treated with lenvatinib, the only HRQoL data available in the literature are derived from 2 small real‐world studies in Italian patients. In a study of 20 patients in Italy with RR‐DTC treated with lenvatinib (10–24 mg/day), patients demonstrated a decrease in HRQoL, as assessed by the EQ‐5D‐3L and EQ‐VAS instruments, during the first 3 months of lenvatinib treatment.^17^ At 1 year after treatment onset, median HRQoL values had returned to levels similar to baseline. Another study assessed the HRQoL of Italian patients with RR‐DTC treated with a starting dose of lenvatinib 24 mg/day (*n* = 39) using the European Organization for the Research and Treatment of Cancer Quality of Life Questionnaire and the pain VAS.^18^ The only significant change from baseline in HRQoL in this study was an increase in diarrhea symptoms during 6 months of lenvatinib treatment.

In Study 211, respective compliance rates in the lenvatinib 18 mg/day and 24 mg/day starting dose arms were high at baseline (98.7%; 97.3%) and remained high through Week 56 (96.4%; 97.0%), which support the robustness of the data collected. In this HRQoL analysis, no significant differences were seen in either time to first deterioration or time to definitive deterioration; however, some potential patterns can be observed in the TTD data. TTD data are consistent with the hypothesis that time to first deterioration may reflect patients' experience of TEAEs, and time to definitive deterioration may align with disease progression. Time to dose reduction in Study 211 was shorter in the lenvatinib 24 mg starting dose arm compared with the 18 mg starting dose arm,^12^ which follows the same pattern as the shorter time to first deterioration in the lenvatinib 24 mg starting dose arm versus the 18 mg starting dose arm, across all instruments (Figure 2A). In comparison, median PFS was longer in the 24 mg starting dose arm than in the 18 mg arm,^12^ which parallels the time to definitive deterioration data, as measured by EQ‐5D VAS. This could indicate that patients experienced TEAEs after initiating treatment that led to first deterioration events but were able to remain on treatment until they experienced definitive deterioration, which could be caused by disease progression. However, numbers of patients with events are small so these data should be interpreted with caution.

Although some TEAEs are known to negatively impact HRQoL outcomes, it has been suggested that antitumor efficacy might have a beneficial effect on QoL (i.e., if patients have favorable outcomes with treatment, this may positively affect HRQoL). In this analysis, HRQoL was assessed in patients who had radiologic tumor responses versus those who did not. As expected, results showed that there was a trend toward longer TTD among patients who had objective radiologic responses. For time to first deterioration, the EQ‐5D VAS, EQ‐5D HUI, and the FACT‐G total score all numerically favored patients with radiologic responses. For time to definitive deterioration, the EQ‐5D HUI nominally significantly favored patients with radiologic responses, and the EQ‐5D VAS and the FACT‐G total score both numerically favored radiologic responders.

The analysis of HRQoL outcomes was a secondary objective of Study 211, with no statistical hypotheses prespecified for these outcomes and, therefore, the results must be viewed as descriptive in nature, and all *P* values considered nominal. Recall periods for the instruments were relatively short (“today” for the EQ‐5D‐3L and 7 days for the FACT‐G) and, thus, only represent HRQoL status at selected intervals. Study 211 was also limited by the relatively small sample size and duration of follow‐up. Furthermore, patient discontinuation limited the availability of HRQoL data over time. Finally, we noted that some patients in clinical practice experience negative symptoms while undergoing treatment with tyrosine kinase inhibitors and, therefore, this study's HRQoL data may represent an incomplete picture of patients' experience, particularly among those patients who do not meet clinical trial criteria. Despite these limitations, to the best of our knowledge, this is the largest, double‐blind, randomized, controlled study to date with prospectively collected data regarding treatment with a kinase inhibitor in patients with RR‐DTC that includes HRQoL data.

These results demonstrate that, contrary to common opinions among clinicians, the starting dose of lenvatinib 24 mg/day did not lead to worse HRQoL than the lower dose of lenvatinib 18 mg/day. In the primary analysis, the ORR as of Week 24 was 40.3% in the lenvatinib 18 mg/day starting dose arm versus 57.3% in the 24 mg/day starting dose arm, and the rates of TEAEs with Common Terminology Criteria for Adverse Events grade ≥3 were similar between the arms. Together with the efficacy and safety results,^12^ these HRQoL data from Study 211 support use of the approved lenvatinib 24 mg starting dose to optimize efficacy, safety, and HRQoL for patients with RR‐DTC.

## AUTHOR CONTRIBUTIONS

**Matthew H. Taylor:** Conceptualization (equal); formal analysis (equal); investigation (equal); methodology (equal); supervision (equal); writing – original draft (equal); writing – review and editing (equal). **Sophie Leboulleux:** Investigation (equal); writing – original draft (equal); writing – review and editing (equal). **Yury Panaseykin:** Investigation (equal); writing – original draft (equal); writing – review and editing (equal). **Bhavana Konda:** Investigation (equal); writing – original draft (equal); writing – review and editing (equal). **Christelle De La Fouchardiere:** Investigation (equal); writing – original draft (equal); writing – review and editing (equal). **Brett G.M. Hughes:** Formal analysis (equal); investigation (equal); writing – original draft (equal); writing – review and editing (equal). **Andrew G. Gianoukakis:** Investigation (equal); writing – original draft (equal); writing – review and editing (equal). **Young Joo Park:** Investigation (equal); writing – original draft (equal); writing – review and editing (equal). **Ilia Romanov:** Investigation (equal); writing – original draft (equal); writing – review and editing (equal). **Monika K. Krzyzanowska:** Investigation (equal); writing – original draft (equal); writing – review and editing (equal). **Diana Garibinsky:** Formal analysis (equal); validation (equal); writing – original draft (equal); writing – review and editing (equal). **Bintu Sherif:** Formal analysis (equal); validation (equal); writing – original draft (equal); writing – review and editing (equal). **Jie Janice Pan:** Formal analysis (equal); validation (equal); writing – original draft (equal); writing – review and editing (equal). **Terri A. Binder:** Conceptualization (equal); formal analysis (equal); methodology (equal); supervision (equal); writing – original draft (equal); writing – review and editing (equal). **Nicholas Sauter:** Formal analysis (equal); validation (equal); writing – original draft (equal); writing – review and editing (equal). **Ran Xie:** Formal analysis (equal); validation (equal); writing – original draft (equal); writing – review and editing (equal). **Marcia S Brose:** Conceptualization (equal); formal analysis (equal); investigation (equal); methodology (equal); supervision (equal); writing – original draft (equal); writing – review and editing (equal).

## FUNDING INFORMATION

This study was funded by Eisai Inc., and Merck Sharp & Dohme LLC, a subsidiary of Merck & Co., Inc. Eisai Inc. participated in the design and conduct of the study, data management, and data analysis. Eisai Inc. participated in the interpretation of data in conjunction with the investigators, and in the preparation, review, and approval of the manuscript. The authors had full access to the data and control of the final approval and decision to submit the manuscript for publication.

## CONFLICT OF INTEREST

Matthew H. Taylor: Consulting/advisory board member (honoraria paid to MHT): Bristol Myers Squibb, Eisai Inc., Novartis, Merck, Pfizer, Bayer, Sanofi/Genzyme, Regeneron, Bayer, Array Biopharma, LOXO Oncology, Blueprint Medicines, Immuneonc, Exelixis, Cascade Prodrug. Speakers' bureau (honoraria paid to MHT): Bristol Myers Squibb, Eisai Inc., Blueprint Medicines, Merck. Research Funding (all funding to MHT's institution): Bristol Myers Squibb, Merck Sharp & Dohme Corp., Pharmacyclics, AstraZeneca, Eisai, Incyte, EMD Serono, Novartis, Seattle Genetics, AbbVie, Genentech, Eli Lilly, Roche, Acerta Pharma, Genzyme Corporation, Pfizer.

Sophie Leboulleux: Advisory Board: Bayer, Eisai, Lilly; Invited Speaker: Eisai.

Yury Panaseykin: Nothing to disclose.

Bhavana Konda: Research funding (all funding to institution): Eisai, Merck, Bristol Myers Squibb, Xencor, Eli Lilly & Co.

Christelle de La Fouchardiere: Honoraria: Eisai, Roche, Servier, Amgen, Bayer, Pierre Fabre Oncologie, Bristol Myers Squibb; Non‐financial support: Roche, Servier, Amgen, Bayer, Pierre Fabre Oncologie, Bristol Myers Squibb.

Brett G.M. Hughes: Advisory Board: MSD, BMS, Roche, Pfizer, AZ, Eisai, Takeda, Sanofi; Grant (Institutional): Amgen.

Andrew G. Gianoukakis: Advisory Board: Eisai, Exelixis.

Young Joo Park: Consultant (Steering committee): Novartis; Research funding: Lilly.

Ilia Romanov: Honoraria: Eisai, Bristol Myers Squibb, Merck Serono.

Monika K. Krzyzanowska: Advisory Board: Eisai, Ipsen, Novartis; Research funding: Ipsen, Eisai.

Diana Garbinsky is a full‐time employee of RTI Health Solutions.

Bintu Sherif is a full‐time employee of RTI Health Solutions.

Jie Janice Pan is an employee of Eisai Inc.

Terri A. Binder is a former employee of, and current consultant to, Eisai Inc., and an Amgen Inc. shareholder.

Nicholas Sauter is an employee of Eisai Inc.

Ran Xie is an employee of Eisai Inc.

Marcia S. Brose: Consulting/advisory board member (honoraria paid to MSB): Eisai Inc, Novartis, Bayer, Sanofi/Genzyme, LOXO Oncology, Blueprint Medicines, Exelixis; Research Funding (all funding to MSB's institution): Eisai, Novartis, Genentech, Eli Lilly, Roche.

## Supporting information


Data S1

## Data Availability

The data will not be available for sharing at this time because the data are commercially confidential. However, Eisai will consider written requests to share the data on a case‐by‐case basis.
