# Supplementary material for: Health‐related quality‐of‐life analyses from a multicenter, randomized, double‐blind phase 2 study of patients with differentiated thyroid cancer treated with lenvatinib 18 or 24 mg/day
Source: Cancer Med. 2022 Dec 4;12(4):4332–42. doi: 10.1002/cam4.5308 (PMC9972135; doi:10.1002/cam4.5308)
Supplement: Supplementary file 1 — Data S1 [file CAM4-12-4332-s001.docx]

**Online only supplementary information**

**METHODS**

*Study 211 Efficacy and Safety Assessments*

Patients received lenvatinib in 28-day cycles and underwent tumor assessments using RECIST v1.1 every 8 weeks until documentation of disease progression, another anticancer therapy was initiated, or the data cutoff for the primary analysis. Safety assessments consisted of monitoring and recording all AEs, as graded using Common Terminology Criteria for Adverse Events v4.03. Dose reductions were allowed for intolerable grade 2 or greater severity AEs per the study protocol.

*HRQoL Analysis Population*

All HRQoL analyses were based on the full analysis set (ie, all randomly assigned patients). The analysis population included all participants in the full analysis set with valid HRQoL outcome data (ie, participants who completed an HRQoL instrument at a given timepoint).

*Patient-Reported Outcome Instruments*

The EuroQol 5-dimension 3-level scale (EQ-5D-3L) and Functional Assessment of Cancer Therapy–General (FACT-G) instruments are summarized in the table below. Validated translations of the instruments were used across the international regions. On the EQ-5D-3L, patients are asked to indicate their health state by ticking the box next to the most appropriate statement in each of the 5 dimensions. This decision results in a 1-digit number that expresses the level selected for that dimension. Digits for the 5 dimensions are then combined to create a 5-digit number that describes that patient’s health status.

| EQ-5D-3L Instrument | FACT-G Instrument |
| --- | --- |
| Subscales are HUI and VAS | Includes a total score and 4 subscales |
| - 5 Dimensions   1. Mobility   2. Self-care   3. Usual activities   4. Pain/discomfort   5. Anxiety/depression - 3 Levels   1. No problems   2. Some problems   3. Extreme problems | - Subscales   1. Physical well-being   2. Social/family well-being   3. Functional well-being   4. Emotional well-being |
| - Recall period: “Today” | - Recall period: 7 days |
| HUI is derived from the 5 dimensions using country-specific weights^a^   - - Calculated values range from 1 (perfect health) to < 0 (worst health/death)^b^   VAS measures global health status   - - Scale from 0 to 100, in which 100 is the “best imaginable health state” | - Physical, social/family, and functional well-being scales are measured on a scale of 0 (worst) to 28 (best) - Emotional well-being scale is measured on a scale of 0 (worst) to 24 (best) - Total score is on a scale from 0 (worst) to 108 (best) - Higher values indicate better quality of life |

^a^Country weights used in Study 211 were United Kingdom (patients from Belgium, Germany, Italy, France, and Russia [1]), United States (patients from Australia, Canada, and the United States [2]), and South Korea (patients from South Korea [3]).

^b^The predictive value for HUI using the United States algorithm is -0.109 (worst health) to 1 (perfect health).

Abbreviations: EQ-5D-3L, EuroQol 5-dimension 3-level scale; FACT-G, Functional Assessment of Cancer Therapy–General; HUI, health utilities index; VAS, visual analog scale.

Statistical Methods

*Longitudinal analysis*

Each mixed model included treatment, time, a time-by-treatment interaction term, baseline health-related quality of life (HRQoL) score, and the 2 randomization stratification variables: (1) age group (≤ 65 years vs > 65 years) and (2) Eastern Cooperative Oncology Group performance status (ECOG PS) 0 vs 1 or 2 along with patient-specific random intercept and slope terms. The covariance matrix for these random effects was assumed to be unstructured.

*Time to deterioration*

Time to first deterioration was defined as the number of weeks between randomization and the first deterioration event during the treatment period. Time to definitive deterioration was defined as the number of weeks between randomization and the earliest deterioration event during the treatment period with no subsequent recovery above the deterioration threshold. A deterioration event for a HRQoL outcome was defined as a detrimental change in score relative to baseline that exceeded the minimally important difference (MID) threshold for decline for that score. MID thresholds in this analysis were: a decrease of 7 points or more (FACT-G total [4, 5]), decrease of 0.08 points or more (EQ-5D) [6], and decrease of 7 points or more (EQ-VAS) [6]. Death was considered a deterioration event if it occurred within 30 days of the last HRQoL assessment. Patients without a deterioration event who were still alive or who died more than 30 days after their last HRQoL assessment were censored at the date of their last HRQoL assessment.

**RESULTS**

**Supplementary Figure 1.** Mean scores over time for (**a**) EQ-5D HUI, (**b**) FACT-G social/family well-being, (**c**) FACT-G emotional well-being, and (**d**) FACT-G functional well-being.


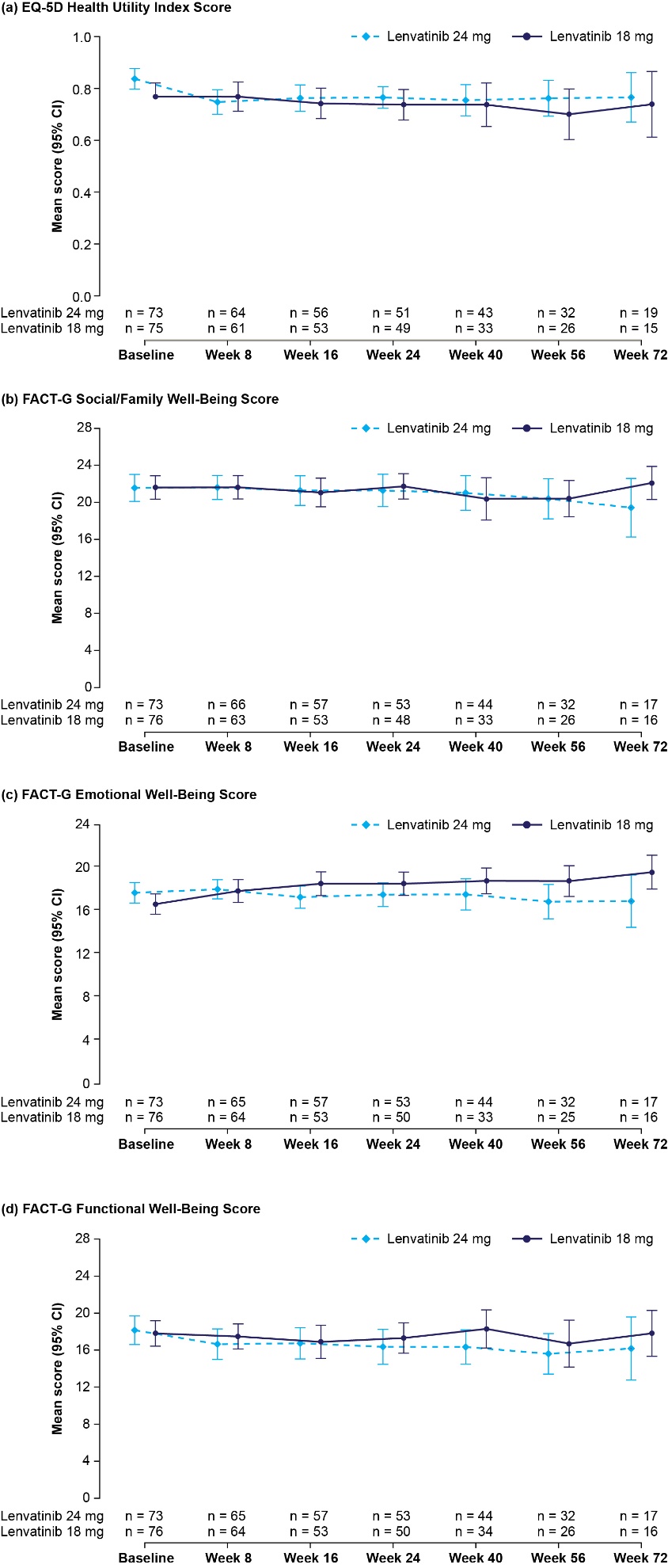


Abbreviations: CI, confidence interval; EQ-5D, EuroQol 5-dimension; FACT-G, Functional Assessment of Cancer Therapy–General.

Supplemental References

- - - 1. Dolan P. Modeling valuations for EuroQol health states. Med Care. 1997;35(11):1095-108.
      2. Shaw JW, Johnson JA, Coons SJ. US valuation of the EQ-5D health states: development and testing of the D1 valuation model. Med Care. 2005;43(3):203-20.
      3. Lee YK, Nam HS, Chuang LH, Kim KY, Yang HK, Kwon IS, Kind P, Kweon SS, Kim YT. South Korean time trade-off values for EQ-5D health states: modeling with observed values for 101 health states. Value Health. 2009;12(8):1187-93.

4. Cella D, Eton DT, Lai JS, Peterman AH, Merkel DE. Combining anchor and distribution-based methods to derive minimal clinically important differences on the Functional Assessment of Cancer Therapy (FACT) anemia and fatigue scales. J Pain Symptom Manage. 2002;24(6):547-61.

5. Cella D, Hahn EA, Dineen K. Meaningful change in cancer-specific quality of life scores: differences between improvement and worsening. Qual Life Res. 2002;11(3):207-21.

6. Pickard AS, Neary MP, Cella D. Estimation of minimally important differences in EQ-5D utility and VAS scores in cancer. Health Qual Life Outcomes. 2007;5:70.
